# Felodipine attenuates neuroinflammatory responses and tau hyperphosphorylation through JNK/P38 signaling in tau-overexpressing AD mice

**DOI:** 10.1186/s13041-024-01137-y

**Published:** 2024-09-02

**Authors:** Jeong-Woo Hwang, Jeongha Kim, Jin-Hee Park, Jinhan Nam, Ji-Yeong Jang, Aran Jo, Hyun-ju Lee, Hyang-Sook Hoe

**Affiliations:** 1 Department of Neural Development and Disease, Korea Brain Research Institute (KBRI), 61, Cheomdan-ro, Dong-gu, Daegu, 41068 Republic of Korea; 2 AI-Based Neurodevelopmental Diseases Digital Therapeutics Group, Korea Brain Research Institute (KBRI), 61, Cheomdan-ro, Dong-gu, Daegu, 41062 Korea; 3 Department of Brain Sciences, Daegu Gyeongbuk Institute of Science & Technology, Daegu, 42988 Korea

**Keywords:** Felodipine, Neuroinflammation, Tau, Microgliosis, Alzheimer's disease

## Abstract

We previously demonstrated that felodipine, an L-type calcium channel blocker, inhibits LPS-mediated neuroinflammatory responses in BV2 microglial cells and wild-type mice. However, the effects of felodipine on tau pathology, a hallmark of Alzheimer's disease (AD), have not been explored yet. Therefore, in the present study, we determined whether felodipine affects neuroinflammation and tau hyperphosphorylation in 3-month-old P301S transgenic mice (PS19), an early phase AD mice model for tauopathy. Felodipine administration decreased tauopathy-mediated microglial activation and NLRP3 expression in PS19 mice but had no effect on tauopathy-associated astrogliosis. In addition, felodipine treatment significantly reduced tau hyperphosphorylation at S202/Thr205 and Thr212/Ser214 residues via inhibiting JNK/P38 signaling in PS19 mice. Collectively, our results suggest that felodipine significantly ameliorates tau hyper-phosphorylation and tauopathy-associated neuroinflammatory responses in AD mice model for tauopathy and could be a novel therapeutic agent for AD.

**Supplementary Information:**

The online version contains supplementary material available at 10.1186/s13041-024-01137-y.

## Main text

The growing size of the older population is increasing the societal burden of Alzheimer's disease (AD), a degenerative brain disease [1]. Accumulating evidence suggests that abnormal regulation of calcium ion (Ca^2+^) channels is involved in development of neurovegetative disease [2, 3]. In particular, the failure of L-type calcium channels (LTCCs) is linked to aging and AD [4] and calcium imbalance can promote neurofibrillary tangle (NFT) formation and Aβ deposition [5]. The potent L-type calcium channel (LTCC) blocker felodipine is an FDA-approved drug for treatment of hypertension [6]. Interestingly, we recently found that felodipine significantly alleviates lipopolysaccharide (LPS)-evoked microglial activation, proinflammatory cytokine production, and spatial memory deficits in vitro and/or wild-type mice [7]. However, the effects of felodipine on tau pathology and tau-mediated neuroinflammatory responses have not been explored in a mouse model of AD.

**Fig. 1:**
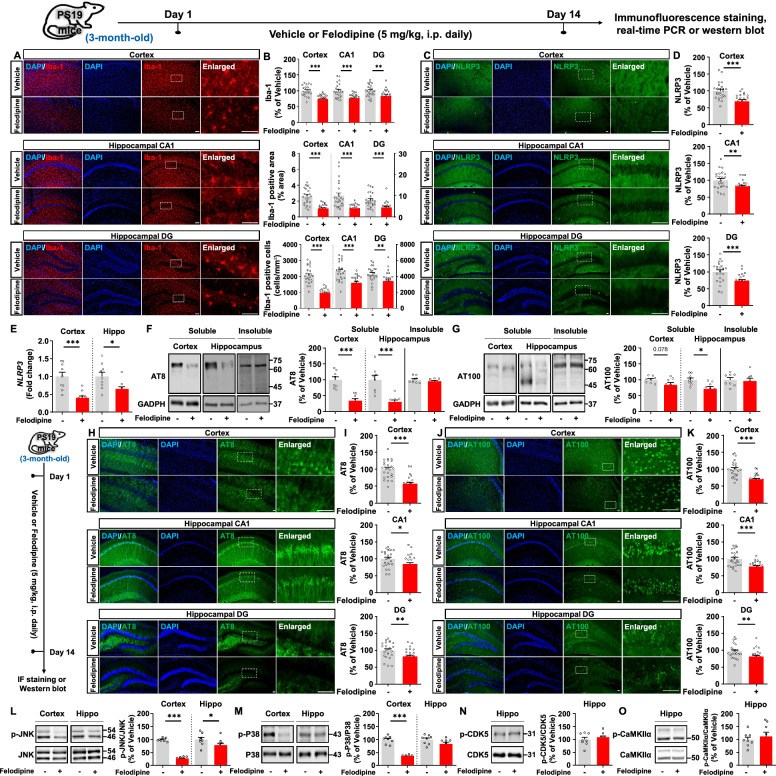
Felodipine treatment signficantly suppresses microgliosis, NLRP3 expression and tau hyperphosphorylation by regulating JNK/P38 signaling in Tau Tg PS19 mice. Three-month-old PS19 mice were injected with vehicle (5% DMSO + 5% PEG + 5% Tween20 + 85% D.W., i.p.) or felodipine (5 mg/kg, i.p.) daily for 14 days. **A**, **C** Immunofluorescence staining was performed with anti-Iba-1 and anti-NLRP3 antibodies. **B** Quantification of data in A (n = 23–24 brain slices from 6 mice/group). **D** Quantification of data in C (n = 24 brain slices from 6 mice/group). **E** The relative mRNA levels of the indicated genes were analyzed by real-time PCR (n = 9–10/group). **F**–**G** Western blotting of RIPA-soluble/insoluble brain lysates was conducted with anti-AT8 and anti-AT100 antibodies (n = 8 mice/group). **H**, **J** Immunofluorescence staining was performed with anti-AT8 and anti-AT100 antibodies. **(I)** Quantification of data in H (n = 24 brain slices from 6 mice/group). **K** Quantification of data in J (n = 23–24 brain slices from 6 mice/group). **L**–**O** Western blotting of brain lysates was conducted with anti-p-JNK, anti-JNK, anti-p-P38, anti-P38, anti-p-CDK5, anti-CDK5, anti-p-CaMKIIα, and anti-CaMKIIα antibodies (n = 8 mice/group). *p < 0.05, **p < 0.01, ***p < 0.001. Scale bar = 100 µm

In the present study, we investigated the effects of felodipine on neuroinflammation and tau hyperphosphorylation and its mechanisms of action in P301S transgenic mice (PS19), a model of AD overexpressing human mutant tau. To test this, Tau Tg PS19 mice were injected with vehicle (5% DMSO + 5% PEG + 5% Tween20 + 85% D.W., i.p.) or felodipine (5 mg/kg, i.p.) daily for 14 days, and immunofluorescence (IF) staining was conducted with an anti-Iba-1 and anti-GFAP antibody. Felodipine treatment significantly reduced Iba-1 fluorescence intensity, Iba-1-labeled area and the number of Iba-1-positive cells (Fig. 1A-B). However, felodipine injection did not alter GFAP fluorescence intensity in Tau Tg PS19 mice (Supplementary Fig. 1). These data suggest that felodipine administration suppresses tauopathy-mediated microgliosis in Tau Tg PS19 mice but not astrogliosis.

NLRP3 is an important molecular target for inhibiting neuroinflammatory responses [8]. Activation of NLRP3 expression results in upregulation of IL-1β, which induces NLRP3 inflammasome complex formation and accelerates AD progression [9]. The NLRP3 inflammasome activates Aβ-induced tau pathology and neurodegeneration in vivo [10, 11]. Activation of NLRP3 inflammasome requires Ca^2+^ signaling, which leads to IL-1β secretion. Interestingly, we recently found that injection of the L- and T-type calcium channel blocker lomerizine significantly inhibits LPS-induced NLRP3 expression in wild-type mice [12]. In this study, we thus examined whether the L-type calcium channel blocker felodipine modulates NLRP3 expression in a mouse model of AD. For this experiments, Tau Tg PS19 mice were injected with felodipine (5 mg/kg, i.p.) or vehicle daily for 14 days, and IF staining was performed with an anti-NLRP3 antibody. Felodipine administration significantly decreased NLRP3 fluorescence intensity in Tau Tg PS19 mice (Fig. 1C–D). In addition, felodipine treatment decreased NLRP3 mRNA levels in the cortex and hippocampus region of Tau Tg PS19 mice (Fig. 1E), suggesting that felodipine treatment may downregulate tauopathy-associated neuroinflammatory responses by inhibiting NLRP3 expression. However, we did not determine whether felodipine treatment regulates the NLRP3 inflammasome complex formation. Thus, it is possible that felodipine-treated Tau Tg PS19 mice may suppresses neuroinflammatory responses by regulating NLRP3 inflammasome complex formation. Other possibility is that felodipine may regulates other neuroinflammation-associated molecular targets to regulate neuroinflammatory responses in Tau Tg PS19 mice, thus we will address in a future study.

Tau hyperphosphorylation is a hallmark of AD and a major target of efforts to develop AD drugs. Abnormal phosphorylation of tau leads to the formation of NFTs, aggregates of hyperphosphorylated tau [13]. The association between calcium channels and tau was first suggested by reports that okadaic acid, a phosphatase inhibitor, activates LTCCs and increases tau phosphorylation [14, 15]. Here, we therefore investigated the effects of felodipine treatment on tau hyperphosphorylation in RIPA-soluble and RIPA-insoluble fractionation of cortex and hippocampus from Tau Tg PS19 mice. We found that felodipine treatment significantly reduced RIPA-soluble tau hyperphosphorylation at Ser202/Thr205 (AT8) and Thr212/Ser214 (AT100) residues in the cortex and hippocampus regions, but not RIPA-insoluble tau levels (Fig. 1F–G). To further confirm our findings as above, we conducted IF staining and found that felodipine-treated Tau Tg PS19 mice significantly decreased tau hyperphosphorylation at Ser202/Thr205 (AT8) and Thr212/Ser214 (AT100) in the cortex and hippocampus (Fig. 1H–K), suggesting that felodipine regulates tauopathy in early phase AD mice model. In this study, we did not examine whether felodipine administration inhibits NFT formation or whether felodipine reduces tau hyperphosphorylation in an aged Tau Tg PS19 mice. Thus, we will investigate the effects of felodipine on tau hyperphosphorylation and/or NFT formation in aged Tau Tg PS19 mice.

Tau kinase activity and JNK/P38 signaling are associated with hyperphosphorylation of tau [16]. Therefore, inhibiting tau kinase activity or JNK/P38 signaling is involved in suppressing tau inclusion therefore being an therapeutic strategy for AD treatment [17]. To address this, we investigated the effects of felodipine on JNK/P38 signaling and found that felodipine-treated Tau Tg PS19 mice significantly downregulated JNK phosphorylation in cortex and hippocampus (Fig. 1L). In addition, felodipine-treated PS19 mice showed decreased P38 phosphorylation in cortex but not in hippocampus (Fig. 1M). However, felodipine did not alter phosphorylation of tau kinases including CDK5 and CaMKIIα in hippocampus of PS19 mice (Fig. 1N–O). These data suggest that felodipine alleviates tauopathy by inhibiting JNK/P38 signaling in Tau Tg PS19 mice. Of course, it is possible that felodipine-treated Tau Tg PS19 mice modulate other tau kinases (i.e., DYRK1A and GSK3β) to alter tau pathology in a mouse model of AD. In future work, we will explore whether felodipine regulates tauopathy in L-type calcium channer (on target)-dependent manner by using AAV shRNA knockdown vector system in AD mice model. In addition, we will investigate the effect of felodipine on various AD pathologies including synaptic loss, mitochondrial dysfunction, autophagy malfunction, metal dyshomeostasis, hormonal imbalance, and oxidative stress in AD mice model. Furthermore, we will assess how the regulatory effect of felodipine on these AD pathologies affect cognitive function via multiple behavioral tests such as Y maze, novel object recognition test, passive avoidance test, and fear conditioning test in AD mice model.

In conclusion, we demonstrated that administration of felodipine, an LTCC blocker, inhibits tauopathy-mediated microglial activation and neuroinflammation-associated molecular target NLRP3 expression in Tau Tg PS19 mice. Importantly, felodipine treatment significantly reduced tau inclusion by suppressing JNK/P38 phosphorylation in Tau Tg PS19 mice. Collectively, our data suggest that felodipine treatment alleviates neuroinflammatory responses and tau pathology in a mouse model of AD.

### Supplementary Information

Supplementary material 1

## Data Availability

All data generated and/or analyzed during this study are included in this published article and its supplementary materials. Materials and methods are presented in the supplementary materials.

## Abbreviations

AD: Alzheimer’s disease
LTCCs: L-type calcium channels
CDK: Cyclin-dependent kinase
GSK3β: Glycogen synthesis kinase 3 beta
JNK: C-Jun N-terminal kinase
CaMKIIα: Calcium/calmodulin-dependent protein kinase II α

## References

[1] Scheltens P, et al. Alzheimer’s disease. Lancet. 2021;397(10284):1577–90. 33667416 10.1016/S0140-6736(20)32205-4 PMC8354300

[2] Ortner NJ, Striessnig J. L-type calcium channels as drug targets in CNS disorders. Channels (Austin). 2016;10(1):7–13. 26039257 10.1080/19336950.2015.1048936 PMC4802752

[3] Siddiqi FH, et al. Felodipine induces autophagy in mouse brains with pharmacokinetics amenable to repurposing. Nat Commun. 2019;10(1):1817. 31000720 10.1038/s41467-019-09494-2 PMC6472390

[4] Crossley CA, Rajani V, Yuan Q. Modulation of L-type calcium channels in Alzheimer’s disease: a potential therapeutic target. Comput Struct Biotechnol J. 2023;21:11–20. 36514335 10.1016/j.csbj.2022.11.049 PMC9719069

[5] Etcheberrigaray R, et al. Calcium responses in fibroblasts from asymptomatic members of Alzheimer’s disease families. Neurobiol Dis. 1998;5(1):37–45. 9702786 10.1006/nbdi.1998.0176

[6] Walton T, Symes LR. Felodipine and isradipine: new calcium-channel-blocking agents for the treatment of hypertension. Clin Pharm. 1993;12(4):261–75. 8458178

[7] Kim J, et al. L-Type Ca(2+) channel inhibition rescues the LPS induced neuroinflammatory response and impairments in spatial memory and dendritic spine formation. Int J Mol Sci. 2022;23(21):13606. 36362394 10.3390/ijms232113606 PMC9655622

[8] Swanson KV, Deng M, Ting JP. The NLRP3 inflammasome: molecular activation and regulation to therapeutics. Nat Rev Immunol. 2019;19(8):477–89. 31036962 10.1038/s41577-019-0165-0 PMC7807242

[9] Liang T, et al. The role of NLRP3 inflammasome in alzheimer’s disease and potential therapeutic targets. Front Pharmacol. 2022. 10.3389/fphar.2022.845185. 35250595 10.3389/fphar.2022.845185 PMC8889079

[10] Nakanishi A, et al. Amyloid β directly interacts with NLRP3 to initiate inflammasome activation: identification of an intrinsic NLRP3 ligand in a cell-free system. Inflam Regener. 2018;38(1):27. 10.1186/s41232-018-0085-6 PMC6231249 30459926

[11] Stancu IC, et al. The NLRP3 inflammasome modulates tau pathology and neurodegeneration in a tauopathy model. Glia. 2022;70(6):1117–32. 35174546 10.1002/glia.24160 PMC9307007

[12] Park J-H, et al. Lomerizine inhibits LPS-mediated neuroinflammation and tau hyperphosphorylation by modulating NLRP3, DYRK1A, and GSK3α/β. Front Immunol. 2023. 10.3389/fimmu.2023.1150940. 37435081 10.3389/fimmu.2023.1150940 PMC10331167

[13] Medeiros R, Baglietto-Vargas D, LaFerla FM. The role of tau in Alzheimer’s disease and related disorders. CNS Neurosci Ther. 2011;17(5):514–24. 20553310 10.1111/j.1755-5949.2010.00177.x PMC4072215

[14] Metin-Armağan D, et al. Okadaic acid-induced tau hyperphosphorylation and the downregulation of Pin1 expression in primary cortical neurons. J Chem Neuroanat. 2018;92:41–7. 29860071 10.1016/j.jchemneu.2018.05.006

[15] Zhang Z, Simpkins JW. Okadaic acid induces tau phosphorylation in SH-SY5Y cells in an estrogen-preventable manner. Brain Res. 2010;1345:176–81. 20457142 10.1016/j.brainres.2010.04.074 PMC2913890

[16] Guo T, Noble W, Hanger DP. Roles of tau protein in health and disease. Acta Neuropathol. 2017;133(5):665–704. 28386764 10.1007/s00401-017-1707-9 PMC5390006

[17] Cao M, et al. Effect of c-Jun N-terminal kinase (JNK)/p38 mitogen-activated protein kinase (p38 MAPK) in morphine-induced tau protein hyperphosphorylation. Behav Brain Res. 2013;237:249–55. 23026374 10.1016/j.bbr.2012.09.040

