# Supplementary material for: Felodipine attenuates neuroinflammatory responses and tau hyperphosphorylation through JNK/P38 signaling in tau-overexpressing AD mice
Source: Mol Brain. 2024 Sep 2;17:62. doi: 10.1186/s13041-024-01137-y (PMC11367747; doi:10.1186/s13041-024-01137-y)
Supplement: Supplementary file 1 — Supplementary material 1 [file 13041_2024_1137_MOESM1_ESM.docx]

**Felodipine attenuates neuroinflammatory responses and tau hyperphosphorylation through JNK/P38 signaling in tau-overexpressing AD mice**

Jeong-Woo Hwang^1,2,4^| Jeongha Kim^1,2,4^| Jin-Hee Park^1,4^| Jinhan Nam ^1^| Ji-Yeong Jang^1,2,3^| Aran Jo^1,2^| Hyun-ju Lee^1^,^2,*^| Hyang-Sook Hoe^1,2,3,^*

^1^Department of Neural Development and Disease, Korea Brain Research Institute (KBRI), 61, Cheomdan-ro, Daegu, Republic of Korea; ^2^AI-based neurodevelopmental diseases digital therapeutics group; ^3^Department of Brain Sciences, Daegu Gyeongbuk Institute of Science & Technology, Daegu 42988, Korea. ^4^These authors contributed equally to this work.

*Corresponding author

**Hyang-Sook Hoe, Ph.D**.: Department of Neural Development and Disease, Korea Brain Research Institute (KBRI), 61 Cheomdan-ro, Dong-gu, Daegu, Korea, 41068; E-mail: [sookhoe72@kbri.re.kr](mailto:sookhoe72@kbri.re.kr)

**Hyun-ju Lee, Ph.D**.: Department of Neural Development and Disease, Korea Brain Research Institute (KBRI), 61 Cheomdan-ro, Dong-gu, Daegu, Korea, 41068; E-mail: [hjlee@kbri.re.kr](mailto:hjlee@kbri.re.kr)

**
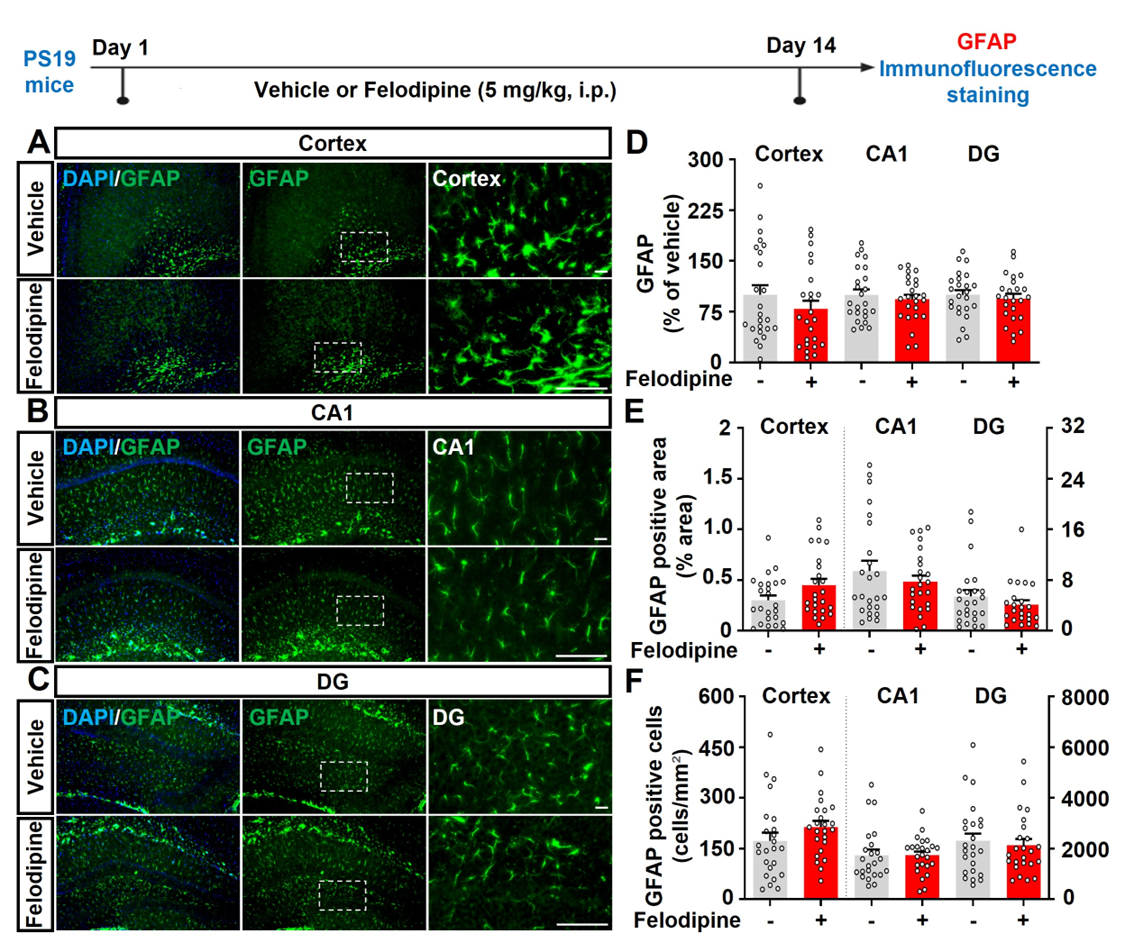
**

**Supplementary Fig 1. Felodipine administration does not affect astrocyte activation in tau-overexpressing (Tau Tg) PS19 mice. (A-C)** Immunofluorescence staining of GFAP in brain tissues from 3-month-old Tau Tg PS19 mice injected with vehicle (5% DMSO + 5% PEG + 5% Tween20 + 85% D.W.) or felodipine (5 mg/kg, i.p.) daily for 14 days. (**D-F)** Quantification of data in A-C (n = 24 brain slices from 6 mice/group). Scale bar = 100 µm.

**Materials and methods**

**Ethics statement**

All experimental procedures were approved by the institutional biosafety committee (IBC) and were performed in accordance with approved animal protocols and guidelines of the Korea Brain Research Institute (KBRI, approval no. IACUC-22-00046).

**Felodipine treatment**

Felodipine (Cat. No. F0814; Tokyo Chemical Industry, Tokyo, Japan) was dissolved in vehicle (5% DMSO + 5% PEG + 5% Tween20 + 85% DW) and intraperitoneally (i.p.) injected daily at a dose of 5 mg/kg for 14 days in 3-month-old male Tau Tg PS19 mice.

**Human mutant Tau-overexpressing P301S transgenic mice (PS19)**

The effects of felodipine on tau pathology and tau-mediated neuroinflammatory response were examined in 3-month-old male Tau Tg PS19 mice. Male mice were used to minimize the effects of hormones. The mice were maintained in a pathogen-free facility with a 12-h photoperiod in cages housing 3-4 mice each and had access to food and water *ad libitum*.

**Immunofluorescence (IF) staining**

Tau Tg PS19 mice were injected with vehicle or felodipine (5 mg/kg, i.p.) daily for 14 days. After the treatment regimen was complete, the brains were dissected and post-fixed in 4% PFA overnight at 4 °C. Next, the brains were immersed in 30% sucrose solution for 3 days at 4 °C for cryoprotection. Finally, the brains were embedded in OCT compound (Sakura Fineetek USA, Torrance, CA) and sliced to a thickness of 30 μm with a cryostat (Leica Biosystems, Buffalo Grove, IL, USA) at -20°C. For IF staining, the brain sections were washed 3 times with 0.2% PBST (PBS + 0.2% Triton-X100) and blocked with 10% normal goat serum (Vector Laboratories, S-1000-20, Burlingame, CA) in PBST for 2 h at room temperature. Next, the brain sections were incubated with primary antibodies (Table 1) at 4°C for 24–72 h. After washing with 0.2% PBST, the brain sections were incubated with fluorescent goat anti-rabbit, anti-rat or anti-mouse secondary antibodies (Table 1) conjugated with Alexa Fluor 488 or 594 for 2 h at room temperature. Finally, the brain sections were washed with PBST, PBST/DAPI, and PBS and mounted on glass slides in mounting solution containing DAPI (Vector Laboratories, H-1200-10, Burlingame, CA). Images of the immunostained tissues were taken with a fluorescence microscope (DMi8, Leica Microsystems).

**Quantification of IF-stained brain tissue**

To quantify the fluorescence intensity in DAPI-stained images of the cortex and hippocampal CA1 and DG regions of the brains of Tau Tg PS19 mice, regions of interest (ROIs) were first selected. Then, the fluorescence intensity of IF staining of Iba-1, GFAP, NLRP3, AT8, and AT100 in each ROI was measured semi-automatically using ImageJ software (version 1.53a, US National Institutes of Health, Bethesda, MD, USA).To measure Iba-1- and GFAP-labeled regions, the cortical and hippocampal CA1 and DG regions were selected as the ROIs in DAPI-stained images, and immunoreactivity thresholds were specified. The percentage immunopositive area of the selected regions was then calculated.

**Real-time PCR**

For real-time PCR, mRNA was extracted from Tau Tg PS19 mice injected with vehicle or felodipine (5mg/kg, i.p.) daily for 14 days using TRI Reagent (Molecular Research Center, Cincinnati, OH, USA). cDNA was synthesized by the Superscript cDNA Premix Kit II with oligo (dT) primers (GeNetBio, Chungman, Korea). The synthesized cDNA was then used as the template in real-time PCR with Fast SYBR Green Master Mix (Thermo Fisher Scientific, Waltham, MA, USA) in a QuantStudio 5 Real-Time PCR System (Applied Biosystems, Thermo Fisher Scientific, Waltham, MA, USA). The primer sequences for real-time PCR are given in Table 2. The cycle threshold (Ct) values were normalized to the value for *gapdh*, and the fold change was calculated relative to the control.

**RIPA-soluble and RIPA-insoluble fractionation**

To investigate whether felodipine regulates soluble and insoluble tau protein levels, Tau Tg PS19 mice were injected with vehicle (5% DMSO + 5% PEG + 5% Tween20 + 85% D.W., i.p.) or felodipine (5mg/kg, i.p.) daily for 14 days. After last treatment, the mice were sacrificed, and the cortex and hippocampus were dissected. The obtained brain tissues were homogenized with RIPA lysis buffer (Merck Millipore, Billerica, MA, USA) containing 1% protease and phosphatase inhibitor cocktail (Thermo Scientific, Waltham, MA, USA) for 2 h at 120 rpm on ice. The brain lysate was centrifuged at 12000rom for 15 min at 4 °C, and the supernatant was collected as the RIPA-soluble fraction stored at -80 °C until analysis. The pellet was washed with RIPA buffer containing with 1 M sucrose, resuspended in 2% SDS, incubated at room temperature for 1 h and centrifuged at 12000rpm for 1min at room temperature. The supernatant was collected as the RIPA-insoluble fraction stored at -80 °C until analysis. For the western blot analysis, 25 µg of protein from the RIPA-soluble or RIPA-insoluble fraction was boiled at 100 °C for 5 min, loaded onto an 8% SDS-page gel, and performed by electrophoresis to protein separation. The proteins were electrotransferred to a PVDF membrane (Millipore, Bedford, MA, USA), then blocked in 5% skim milk or 5% BSA for 1 h and incubated with primary antibody at 4 °C for overnight. Next day, the membrane was incubated with HRP-conjugated goat anti-mouse or anti-rabbit IgG (Enzo Life Sciences, Farmingdale, NY, USA) for 1 h. ECL solution was used to detect protein bands, and the images were acquired and analyzed Fusion Capt Advance software.

**Western blotting (WB)**

For western blotting, Tau Tg PS19 mice injected with vehicle (5% DMSO + 5% PEG + 5% Tween20 + 85% D.W., i.p.) or felodipine (5mg/kg, i.p.) daily for 14 days were anesthetized and cortical/hippocampal tissue were lysed in RIPA buffer (Merck Millipore, Burlington, MA, USA). After lysis, lysates were incubated on ice for 1 h and centrifuged for 15 min at 12,000 rpm three times. Protein concentration in the supernatant was estimated relative to a standard BSA solution, 20-30 μg of protein was separated by sodium dodecyl sulfate-polyacrylamide gel electrophoresis (SDS-PAGE) on an 8% acrylamide gel. The proteins were transferred to a polyvinylidene difluoride (PVDF) membrane (Merck Millipore, Burlington, MA, USA), which was blocked with 5% skim milk or 5% BSA at room temperature for 1 h before incubation overnight with anti-AT8 (1:5000, MN1020, Invitrogen, Waltham, MA, USA), anti-AT100 (1:1000, MN1060, Invitrogen, Waltham, MA, USA), anti-p-JNK (1:1000, 9251s, Cell Signaling Technology, Danvers, MA, USA), anti-JNK (1:1000, SC-7345, Santa Cruz, Dallas, TE, USA), anti-p-P38 (1:1000, 9211s, Cell Signaling Technology, Danvers, MA, USA), anti-P38 (1:1000, 9212, Cell Signaling Technology, Danvers, MA, USA), anti-p-CDK5 (1:500, MBS-9601140, MyBioSource, San Diego, CA, USA), anti-CDK5 (1:1000, 2506, Cell Signaling Technology, Danvers, MA, USA), anti-p-CaMKIIα (1:1000, 12716S, Cell Signaling Technology, Danvers, MA, USA), anti-CaMKIIα (1:1000, Ab92332, Abcam, Cambridge, UK) at 4 °C. The membrane was subsequently incubated with HRP-conjugated goat anti-mouse or anti-rabbit IgG (Enzo Life Sciences, Farmingdale, NY, USA) for 1 h. Lumigen ECL Ultra (TMA-6, Lumigen, Southfield, MI, USA) was used for detection. The membranes were then stripped, incubated with anti-GAPDH (1:10000, 10494-1-AP, Proteintech Group, Inc, Rosemont, IL, USA) and developed accordingly. For image acquisition and analysis, Fusion Capt Advance software (Vilber Lourmat) was used. The used antibody in these experiments described on Table 1.

**Statistical analysis**

Statistical analysis of raw data was performed using GraphPad Prism 8 software (GraphPad Software, San Diego, CA, USA). For comparison between two groups, student's t test was used, and the results were presented as the mean ± SEM (*p < 0.05, **p < 0.01, ***p < 0.001).

Table 1. List of primary and secondary antibodies used in this study.

| **Primary antibodies** | | | | | | | | |
| --- | --- | --- | --- | --- | --- | --- | --- | --- |
| **Immunogen** | **Host** | **Dilution** | | **Manufacturer** | | | **Cat. no** | **Application** |
| Iba-1 | Rabbit | 1:500 | | Wako | | | 019-19741 | IF |
| NLRP3 | Rabbit | 1:200 | | Novus | | | NBP2-12446 | IF |
| AT8 | Mouse | 1:200/1:5000 | | Invitrogen | | | MN1020 | IF/WB |
| AT100 | Mouse | 1:200/1:1000 | | Invitrogen | | | MN1060 | IF/WB |
| p-JNK | Rabbit | 1:1000 | | Cell Signaling Technology | | | 9251S | WB |
| JNK | Mouse | 1:1000 | | Santa Cruz | | | SC-7345 | WB |
| p-P38 | Rabbit | 1:1000 | | Cell Signaling Technology | | | 9211S | WB |
| P38 | Rabbit | 1:1000 | | Cell Signaling Technology | | | 9212 | WB |
| p-CDK5 | Rabbit | 1:500 | | MyBioSource | | | MBS9601140 | WB |
| CDK5 | Rabbit | 1:1000 | | Cell Signaling Technology | | | 2506 | WB |
| p-CaMKIIα | Rabbit | 1:1000 | | Cell Signaling Technology | | | 12716S | WB |
| CaMKIIα | Rabbit | 1:1000 | | Abcam | | | Ab92332 | WB |
| GFAP | Rat | 1:500 | | Invitrogen | | | 13-0300 | IF |
| GAPDH | Rabbit | 1:10000 | | Proteintech | | | 10494-1-AP | WB |
| **Secondary antibodies** | | | | | | | | |
| **Antibody** | | | **Dilution** | | **Manufacturer** | **Cat. no** | | **Application** |
| Goat anti-mouse IgG, 488 | | | 1:200 | | Invitrogen | A11001 | | IF |
| Goat anti-Rat IgG, 488 | | | 1:200 | | Invitrogen | A11006 | | IF |
| Goat anti-rabbit IgG, 594 | | | 1:200 | | Invitrogen | A11012 | | IF |
| Goat anti-rabbit IgG, HRP | | | 1:10000 | | Enzo | ADI-SAB-300-J | | WB |
| Goat anti-mouse IgG, HRP | | | 1:10000 | | Enzo | BML-SA204-0100 | | WB |

Table 2. List of primer sequence used in this study.

| Gene |  | Sequence |
| --- | --- | --- |
| gapdh | Forward | 5’- TGT GTC CGT CGT GGA TCT GA-3’ |
|  | Reverse | 5’-CCT GCTTCA CCA CCT TCT TGA -3’ |
| nlrp3 | Forward | 5’- TCC ACA ATT CTG ACC CAC AA-3’ |
|  | Reverse | 5’- ACC TCA CAG AGG GTC ACC AC-3’ |

Table 3. Statistical analysis results of the *in vivo* experiments.

| **Figure 1B. Iba-1 Fluorescence intensity - Cortex** |
| --- |
| \| Table Analyzed \| Iba-1 intensity \| \| --- \| --- \| \|  \|  \| \| Column A \| Data Set-A \| \| vs. \| vs. \| \| Column B \| Data Set-B \| \|  \|  \| \| Unpaired t test \|  \| \| P value \| <0.0001 \| \| P value summary \| **** \| \| Significantly different (P < 0.05)? \| Yes \| \| One- or two-tailed P value? \| Two-tailed \| \| t, df \| t=6.245, df=46 \| |
| **Figure 1B. Iba-1 Fluorescence intensity – CA1** |
| \| Table Analyzed \| Iba-1 intensity \| \| --- \| --- \| \|  \|  \| \| Column C \| Data Set-C \| \| vs. \| vs. \| \| Column D \| Data Set-D \| \|  \|  \| \| Unpaired t test \|  \| \| P value \| 0.0001 \| \| P value summary \| *** \| \| Significantly different (P < 0.05)? \| Yes \| \| One- or two-tailed P value? \| Two-tailed \| \| t, df \| t=4.208, df=45 \| |
| **Figure 1B. Iba-1 Fluorescence intensity – DG** |
| \| Table Analyzed \| Iba-1 intensity \| \| --- \| --- \| \|  \|  \| \| Column E \| Data Set-E \| \| vs. \| vs. \| \| Column F \| Data Set-F \| \|  \|  \| \| Unpaired t test \|  \| \| P value \| 0.0042 \| \| P value summary \| ** \| \| Significantly different (P < 0.05)? \| Yes \| \| One- or two-tailed P value? \| Two-tailed \| \| t, df \| t=3.017, df=46 \| |
| **Figure 1B. Iba-1 Positive area – Cortex** |
| \| Table Analyzed \| Iba-1 Positive area \| \| --- \| --- \| \|  \|  \| \| Column A \| Data Set-A \| \| vs. \| vs. \| \| Column B \| Data Set-B \| \|  \|  \| \| Unpaired t test \|  \| \| P value \| <0.0001 \| \| P value summary \| **** \| \| Significantly different (P < 0.05)? \| Yes \| \| One- or two-tailed P value? \| Two-tailed \| \| t, df \| t=6.719, df=46 \| |
| **Figure 1B. Iba-1 Positive area – CA1** |
| \| Table Analyzed \| Iba-1 Positive area \| \| --- \| --- \| \|  \|  \| \| Column C \| Data Set-C \| \| vs. \| vs. \| \| Column D \| Data Set-D \| \|  \|  \| \| Unpaired t test \|  \| \| P value \| <0.0001 \| \| P value summary \| **** \| \| Significantly different (P < 0.05)? \| Yes \| \| One- or two-tailed P value? \| Two-tailed \| \| t, df \| t=4.415, df=45 \| |
| **Figure 1B. Iba-1 Positive area – DG** |
| \| Table Analyzed \| Iba-1 Positive area \| \| --- \| --- \| \|  \|  \| \| Column E \| Data Set-E \| \| vs. \| vs. \| \| Column F \| Data Set-F \| \|  \|  \| \| Unpaired t test \|  \| \| P value \| 0.0002 \| \| P value summary \| *** \| \| Significantly different (P < 0.05)? \| Yes \| \| One- or two-tailed P value? \| Two-tailed \| \| t, df \| t=4.069, df=46 \| |
| **Figure 1B. Iba-1 Positive cells – Cortex** |
| \| Table Analyzed \| Iba-1 positive cells \| \| --- \| --- \| \|  \|  \| \| Column A \| Data Set-A \| \| vs. \| vs. \| \| Column B \| Data Set-B \| \|  \|  \| \| Unpaired t test \|  \| \| P value \| <0.0001 \| \| P value summary \| **** \| \| Significantly different (P < 0.05)? \| Yes \| \| One- or two-tailed P value? \| Two-tailed \| \| t, df \| t=9.339, df=46 \| |
| **Figure 1B. Iba-1 Positive cells – CA1** |
| \| Table Analyzed \| Iba-1 positive cells \| \| --- \| --- \| \|  \|  \| \| Column C \| Data Set-C \| \| vs. \| vs. \| \| Column D \| Data Set-D \| \|  \|  \| \| Unpaired t test \|  \| \| P value \| <0.0001 \| \| P value summary \| **** \| \| Significantly different (P < 0.05)? \| Yes \| \| One- or two-tailed P value? \| Two-tailed \| \| t, df \| t=4.654, df=45 \| |
| **Figure 1B. Iba-1 Positive cells – DG** |
| \| Table Analyzed \| Iba-1 positive cells \| \| --- \| --- \| \|  \|  \| \| Column E \| Data Set-E \| \| vs. \| vs. \| \| Column F \| Data Set-F \| \|  \|  \| \| Unpaired t test \|  \| \| P value \| 0.0038 \| \| P value summary \| ** \| \| Significantly different (P < 0.05)? \| Yes \| \| One- or two-tailed P value? \| Two-tailed \| \| t, df \| t=3.045, df=46 \| |
| **Figure 1D. NLRP3 Fluorescence intensity – Cortex** |
| \| Table Analyzed \| NLRP3 - Cortex \| \| --- \| --- \| \|  \|  \| \| Column A \| Data Set-A \| \| vs. \| vs. \| \| Column B \| Data Set-B \| \|  \|  \| \| Unpaired t test \|  \| \| P value \| <0.0001 \| \| P value summary \| **** \| \| Significantly different (P < 0.05)? \| Yes \| \| One- or two-tailed P value? \| Two-tailed \| \| t, df \| t=4.647, df=46 \| |
| **Figure 1D. NLRP3 Fluorescence intensity – CA1** |
| \| Table Analyzed \| NLRP3 - CA1 \| \| --- \| --- \| \|  \|  \| \| Column A \| Data Set-A \| \| vs. \| vs. \| \| Column B \| Data Set-B \| \|  \|  \| \| Unpaired t test \|  \| \| P value \| 0.0080 \| \| P value summary \| ** \| \| Significantly different (P < 0.05)? \| Yes \| \| One- or two-tailed P value? \| Two-tailed \| \| t, df \| t=2.774, df=46 \| |
| **Figure 1D. NLRP3 Fluorescence intensity – DG** |
| \| Table Analyzed \| NLRP3 - DG \| \| --- \| --- \| \|  \|  \| \| Column A \| Data Set-A \| \| vs. \| vs. \| \| Column B \| Data Set-B \| \|  \|  \| \| Unpaired t test \|  \| \| P value \| 0.0006 \| \| P value summary \| *** \| \| Significantly different (P < 0.05)? \| Yes \| \| One- or two-tailed P value? \| Two-tailed \| \| t, df \| t=3.660, df=46 \| |
| **Figure 1E. NLRP3 Real time PCR – Cortex** |
| \| Table Analyzed \| NLRP3 - Real time PCR \| \| --- \| --- \| \|  \|  \| \| Column A \| Data Set-A \| \| vs. \| vs. \| \| Column B \| Data Set-B \| \|  \|  \| \| Unpaired t test \|  \| \| P value \| 0.0002 \| \| P value summary \| *** \| \| Significantly different (P < 0.05)? \| Yes \| \| One- or two-tailed P value? \| Two-tailed \| \| t, df \| t=4.713, df=17 \| |
| **Figure 1E. NLRP3 Real time PCR – Hippocampus** |
| \| Table Analyzed \| NLRP3 - Real time PCR \| \| --- \| --- \| \|  \|  \| \| Column C \| Data Set-C \| \| vs. \| vs. \| \| Column D \| Data Set-D \| \|  \|  \| \| Unpaired t test \|  \| \| P value \| 0.0140 \| \| P value summary \| * \| \| Significantly different (P < 0.05)? \| Yes \| \| One- or two-tailed P value? \| Two-tailed \| \| t, df \| t=2.737, df=17 \| |
| **Figure 1F. AT8 Western blot Soluble – Cortex** |
| \| Table Analyzed \| AT8 Western blot \| \| --- \| --- \| \|  \|  \| \| Column A \| Data Set-A \| \| vs. \| vs. \| \| Column B \| Data Set-B \| \|  \|  \| \| Unpaired t test \|  \| \| P value \| <0.0001 \| \| P value summary \| **** \| \| Significantly different (P < 0.05)? \| Yes \| \| One- or two-tailed P value? \| Two-tailed \| \| t, df \| t=5.589, df=14 \| |
| **Figure 1F. AT8 Western blot Soluble – Hippocampus** |
| \| Table Analyzed \| AT8 Western blot \| \| --- \| --- \| \|  \|  \| \| Column C \| Data Set-C \| \| vs. \| vs. \| \| Column D \| Data Set-D \| \|  \|  \| \| Unpaired t test \|  \| \| P value \| 0.0006 \| \| P value summary \| *** \| \| Significantly different (P < 0.05)? \| Yes \| \| One- or two-tailed P value? \| Two-tailed \| \| t, df \| t=4.415, df=14 \| |
| **Figure 1F. AT8 Western blot Insoluble – Hippocampus** |
| \| Table Analyzed \| AT8 Western blot \| \| --- \| --- \| \|  \|  \| \| Column E \| Data Set-E \| \| vs. \| vs. \| \| Column F \| Data Set-F \| \|  \|  \| \| Unpaired t test \|  \| \| P value \| 0.3519 \| \| P value summary \| ns \| \| Significantly different (P < 0.05)? \| No \| \| One- or two-tailed P value? \| Two-tailed \| \| t, df \| t=0.9630, df=14 \| |
| **Figure 1G. AT100 Western blot Soluble – Cortex** |
| \| Table Analyzed \| AT100 Western blot \| \| --- \| --- \| \|  \|  \| \| Column A \| Data Set-A \| \| vs. \| vs. \| \| Column B \| Data Set-B \| \|  \|  \| \| Unpaired t test \|  \| \| P value \| 0.0787 \| \| P value summary \| ns \| \| Significantly different (P < 0.05)? \| No \| \| One- or two-tailed P value? \| Two-tailed \| \| t, df \| t=1.897, df=14 \| |
| **Figure 1G. AT100 Western blot Soluble – Hippocampus** |
| \| Table Analyzed \| AT100 Western blot \| \| --- \| --- \| \|  \|  \| \| Column C \| Data Set-C \| \| vs. \| vs. \| \| Column D \| Data Set-D \| \|  \|  \| \| Unpaired t test \|  \| \| P value \| 0.0149 \| \| P value summary \| * \| \| Significantly different (P < 0.05)? \| Yes \| \| One- or two-tailed P value? \| Two-tailed \| \| t, df \| t=2.773, df=14 \| |
| **Figure 1G. AT100 Western blot Insoluble – Hippocampus** |
| \| Table Analyzed \| AT100 Western blot \| \| --- \| --- \| \|  \|  \| \| Column E \| Data Set-E \| \| vs. \| vs. \| \| Column F \| Data Set-F \| \|  \|  \| \| Unpaired t test \|  \| \| P value \| 0.7214 \| \| P value summary \| ns \| \| Significantly different (P < 0.05)? \| No \| \| One- or two-tailed P value? \| Two-tailed \| \| t, df \| t=0.3639, df=14 \| |
| **Figure 1I. AT8 Fluorescence intensity – Cortex** |
| \| \| Table Analyzed \| AT8-Cortex \| \| --- \| --- \| \|  \|  \| \| Column B \| Data Set-B \| \| vs. \| vs. \| \| Column A \| Data Set-A \| \|  \|  \| \| Unpaired t test \|  \| \| P value \| <0.0001 \| \| P value summary \| **** \| \| Significantly different (P < 0.05)? \| Yes \| \| One- or two-tailed P value? \| Two-tailed \| \| t, df \| t=6.089, df=46 \| \|  \| \| --- \| --- \| --- \| --- \| --- \| --- \| --- \| --- \| --- \| --- \| --- \| --- \| --- \| --- \| --- \| --- \| --- \| --- \| --- \| --- \| --- \| --- \| --- \| --- \| --- \| --- \| |
| **Figure 1I. AT8 Fluorescence intensity – CA1** |
| \| Table Analyzed \| AT8-CA1 \| \| --- \| --- \| \|  \|  \| \| Column A \| Data Set-A \| \| vs. \| vs. \| \| Column B \| Data Set-B \| \|  \|  \| \| Unpaired t test \|  \| \| P value \| 0.0308 \| \| P value summary \| * \| \| Significantly different (P < 0.05)? \| Yes \| \| One- or two-tailed P value? \| Two-tailed \| \| t, df \| t=2.229, df=46 \| |
| **Figure 1I. AT8 Fluorescence intensity – DG** |
| \| Table Analyzed \| AT8-DG \| \| --- \| --- \| \|  \|  \| \| Column B \| Data Set-B \| \| vs. \| vs. \| \| Column A \| Data Set-A \| \|  \|  \| \| Unpaired t test \|  \| \| P value \| 0.0078 \| \| P value summary \| ** \| \| Significantly different (P < 0.05)? \| Yes \| \| One- or two-tailed P value? \| Two-tailed \| \| t, df \| t=2.784, df=46 \| |
| **Figure 1K. AT100 Fluorescence intensity – Cortex** |
| \| Table Analyzed \| AT100-Cortex \| \| --- \| --- \| \|  \|  \| \| Column B \| Data Set-B \| \| vs. \| vs. \| \| Column A \| Data Set-A \| \|  \|  \| \| Unpaired t test \|  \| \| P value \| <0.0001 \| \| P value summary \| **** \| \| Significantly different (P < 0.05)? \| Yes \| \| One- or two-tailed P value? \| Two-tailed \| \| t, df \| t=5.165, df=46 \| |
| **Figure 1K. AT100 Fluorescence intensity – CA1** |
| \| Table Analyzed \| AT100-CA1 \| \| --- \| --- \| \|  \|  \| \| Column B \| Data Set-B \| \| vs. \| vs. \| \| Column A \| Data Set-A \| \|  \|  \| \| Unpaired t test \|  \| \| P value \| 0.0002 \| \| P value summary \| *** \| \| Significantly different (P < 0.05)? \| Yes \| \| One- or two-tailed P value? \| Two-tailed \| \| t, df \| t=3.988, df=46 \| |
| **Figure 1K. AT100 Fluorescence intensity – DG** |
| \| Table Analyzed \| AT100-DG \| \| --- \| --- \| \|  \|  \| \| Column B \| Data Set-B \| \| vs. \| vs. \| \| Column A \| Data Set-A \| \|  \|  \| \| Unpaired t test \|  \| \| P value \| 0.0096 \| \| P value summary \| ** \| \| Significantly different (P < 0.05)? \| Yes \| \| One- or two-tailed P value? \| Two-tailed \| \| t, df \| t=2.705, df=45 \| |
| **Figure 1L. p-JNK/JNK Western blot - Cortex** |
| \| \| Table Analyzed \| p-JNK Western blot \| \| --- \| --- \| \|  \|  \| \| Column B \| Data Set-B \| \| vs. \| vs. \| \| Column A \| Data Set-A \| \|  \|  \| \| Unpaired t test \|  \| \| P value \| <0.0001 \| \| P value summary \| **** \| \| Significantly different (P < 0.05)? \| Yes \| \| One- or two-tailed P value? \| Two-tailed \| \| t, df \| t=20.55, df=14 \| \|  \| \| --- \| --- \| --- \| --- \| --- \| --- \| --- \| --- \| --- \| --- \| --- \| --- \| --- \| --- \| --- \| --- \| --- \| --- \| --- \| --- \| --- \| --- \| --- \| --- \| --- \| --- \| |
| **Figure 1L. p-JNK/JNK Western blot – Hippocampus** |
| \| Table Analyzed \| p-JNK Western blot \| \| --- \| --- \| \|  \|  \| \| Column C \| Data Set-C \| \| vs. \| vs. \| \| Column D \| Data Set-D \| \|  \|  \| \| Unpaired t test \|  \| \| P value \| 0.0886 \| \| P value summary \| ns \| \| Significantly different (P < 0.05)? \| No \| \| One- or two-tailed P value? \| Two-tailed \| \| t, df \| t=1.830, df=14 \| |
| **Figure 1M. p-P38/P38 Western blot – Cortex** |
| \| Table Analyzed \| p-P38 Western blot \| \| --- \| --- \| \|  \|  \| \| Column B \| Data Set-B \| \| vs. \| vs. \| \| Column A \| Data Set-A \| \|  \|  \| \| Unpaired t test \|  \| \| P value \| <0.0001 \| \| P value summary \| **** \| \| Significantly different (P < 0.05)? \| Yes \| \| One- or two-tailed P value? \| Two-tailed \| \| t, df \| t=11.35, df=14 \| |
| **Figure 1M. p-P38/P38 Western blot – Hippocampus** |
| \| Table Analyzed \| p-JNK Western blot \| \| --- \| --- \| \|  \|  \| \| Column C \| Data Set-C \| \| vs. \| vs. \| \| Column D \| Data Set-D \| \|  \|  \| \| Unpaired t test \|  \| \| P value \| 0.0443 \| \| P value summary \| * \| \| Significantly different (P < 0.05)? \| Yes \| \| One- or two-tailed P value? \| One-tailed \| \| t, df \| t=1.830, df=14 \| |
| **Figure 1N. p-CDK5/CDK5 Western blot – Hippocampus** |
| \| Table Analyzed \| p-CDK5-Hippocampus \| \| --- \| --- \| \|  \|  \| \| Column B \| Data Set-B \| \| vs. \| vs. \| \| Column A \| Data Set-A \| \|  \|  \| \| Unpaired t test \|  \| \| P value \| 0.5084 \| \| P value summary \| ns \| \| Significantly different (P < 0.05)? \| No \| \| One- or two-tailed P value? \| Two-tailed \| \| t, df \| t=0.6787, df=14 \| |
| **Figure 1O. p-CaMKIIα/CaMKIIα Western blot – Hippocampus** |
| \| Table Analyzed \| p-CaMKII-Hippocampus \| \| --- \| --- \| \|  \|  \| \| Column B \| Data Set-B \| \| vs. \| vs. \| \| Column A \| Data Set-A \| \|  \|  \| \| Unpaired t test \|  \| \| P value \| 0.6108 \| \| P value summary \| ns \| \| Significantly different (P < 0.05)? \| No \| \| One- or two-tailed P value? \| Two-tailed \| \| t, df \| t=0.5206, df=14 \| |
| **Supplementary figure 1D. GFAP fluorescence intensity - Cortex** |
| \| Table Analyzed \| GFAP intensity \| \| --- \| --- \| \|  \|  \| \| Column A \| Data Set-A \| \| vs. \| vs. \| \| Column B \| Data Set-B \| \|  \|  \| \| Unpaired t test \|  \| \| P value \| 0.2751 \| \| P value summary \| ns \| \| Significantly different (P < 0.05)? \| No \| \| One- or two-tailed P value? \| Two-tailed \| \| t, df \| t=1.105, df=46 \| |
| **Supplementary figure 1D. GFAP fluorescence intensity – CA1** |
| \| Table Analyzed \| GFAP intensity \| \| --- \| --- \| \|  \|  \| \| Column C \| Data Set-C \| \| vs. \| vs. \| \| Column D \| Data Set-D \| \|  \|  \| \| Unpaired t test \|  \| \| P value \| 0.5342 \| \| P value summary \| ns \| \| Significantly different (P < 0.05)? \| No \| \| One- or two-tailed P value? \| Two-tailed \| \| t, df \| t=0.6263, df=46 \| |
| **Supplementary figure 1D. GFAP fluorescence intensity - DG** |
| \| Table Analyzed \| GFAP intensity \| \| --- \| --- \| \|  \|  \| \| Column E \| Data Set-E \| \| vs. \| vs. \| \| Column F \| Data Set-F \| \|  \|  \| \| Unpaired t test \|  \| \| P value \| 0.5873 \| \| P value summary \| ns \| \| Significantly different (P < 0.05)? \| No \| \| One- or two-tailed P value? \| Two-tailed \| \| t, df \| t=0.5466, df=46 \| |
| **Supplementary figure 1E. GFAP positive area - Cortex** |
| \| Table Analyzed \| GFAP positive area \| \| --- \| --- \| \|  \|  \| \| Column A \| Data Set-A \| \| vs. \| vs. \| \| Column B \| Data Set-B \| \|  \|  \| \| Unpaired t test \|  \| \| P value \| 0.0682 \| \| P value summary \| ns \| \| Significantly different (P < 0.05)? \| No \| \| One- or two-tailed P value? \| Two-tailed \| \| t, df \| t=1.868, df=46 \| |
| **Supplementary figure 1E. GFAP positive area – CA1** |
| \| Table Analyzed \| GFAP positive area \| \| --- \| --- \| \|  \|  \| \| Column C \| Data Set-C \| \| vs. \| vs. \| \| Column D \| Data Set-D \| \|  \|  \| \| Unpaired t test \|  \| \| P value \| 0.3737 \| \| P value summary \| ns \| \| Significantly different (P < 0.05)? \| No \| \| One- or two-tailed P value? \| Two-tailed \| \| t, df \| t=0.8983, df=46 \| |
| **Supplementary figure 1E. GFAP positive area - DG** |
| \| Table Analyzed \| GFAP positive area \| \| --- \| --- \| \|  \|  \| \| Column E \| Data Set-E \| \| vs. \| vs. \| \| Column F \| Data Set-F \| \|  \|  \| \| Unpaired t test \|  \| \| P value \| 0.2954 \| \| P value summary \| ns \| \| Significantly different (P < 0.05)? \| No \| \| One- or two-tailed P value? \| Two-tailed \| \| t, df \| t=1.059, df=45 \| |
| **Supplementary figure 1F. GFAP positive cells - Cortex** |
| \| Table Analyzed \| GFAP positive cells \| \| --- \| --- \| \|  \|  \| \| Column A \| Data Set-A \| \| vs. \| vs. \| \| Column B \| Data Set-B \| \|  \|  \| \| Unpaired t test \|  \| \| P value \| 0.1836 \| \| P value summary \| ns \| \| Significantly different (P < 0.05)? \| No \| \| One- or two-tailed P value? \| Two-tailed \| \| t, df \| t=1.350, df=46 \| |
| **Supplementary figure 1F. GFAP positive cells – CA1** |
| \| Table Analyzed \| GFAP positive cells \| \| --- \| --- \| \|  \|  \| \| Column C \| Data Set-C \| \| vs. \| vs. \| \| Column D \| Data Set-D \| \|  \|  \| \| Unpaired t test \|  \| \| P value \| 0.9736 \| \| P value summary \| ns \| \| Significantly different (P < 0.05)? \| No \| \| One- or two-tailed P value? \| Two-tailed \| \| t, df \| t=0.03329, df=45 \| |
| **Supplementary figure 1F. GFAP positive cells – DG** |
| \| Table Analyzed \| GFAP positive cells \| \| --- \| --- \| \|  \|  \| \| Column E \| Data Set-E \| \| vs. \| vs. \| \| Column F \| Data Set-F \| \|  \|  \| \| Unpaired t test \|  \| \| P value \| 0.6566 \| \| P value summary \| ns \| \| Significantly different (P < 0.05)? \| No \| \| One- or two-tailed P value? \| Two-tailed \| \| t, df \| t=0.4476, df=46 \| |
